# Recent advances in the development of ^225^Ac- and ^211^At-labeled radioligands for radiotheranostics

**DOI:** 10.1007/s44211-024-00514-w

**Published:** 2024-04-02

**Authors:** Masayuki Munekane, Takeshi Fuchigami, Kazuma Ogawa

**Affiliations:** 1 Graduate School of Medical Sciences, Kanazawa University, Kakuma-Machi, Kanazawa, Ishikawa 920-1192 Japan; 2 Institute for Frontier Science Initiative, Kanazawa University, Kakuma-Machi, Kanazawa, Ishikawa 920-1192 Japan

**Keywords:** Radiotheranostics, Targeted alpha therapy, Cancer, Radiopharmaceuticals, Molecular Imaging

## Abstract

Radiotheranostics utilizes a set of radioligands incorporating diagnostic or therapeutic radionuclides to achieve both diagnosis and therapy. Imaging probes using diagnostic radionuclides have been used for systemic cancer imaging. Integration of therapeutic radionuclides into the imaging probes serves as potent agents for radionuclide therapy. Among them, targeted alpha therapy (TAT) is a promising next-generation cancer therapy. The α-particles emitted by the radioligands used in TAT result in a high linear energy transfer over a short range, inducing substantial damage to nearby cells surrounding the binding site. Therefore, the key to successful cancer treatment with minimal side effects by TAT depends on the selective delivery of radioligands to their targets. Recently, TAT agents targeting biomolecules highly expressed in various cancer cells, such as sodium/iodide symporter, norepinephrine transporter, somatostatin receptor, α_v_β_3_ integrin, prostate-specific membrane antigen, fibroblast-activation protein, and human epidermal growth factor receptor 2 have been developed and have made remarkable progress toward clinical application. In this review, we focus on two radionuclides, ^225^Ac and ^211^At, which are expected to have a wide range of applications in TAT. We also introduce recent fundamental and clinical studies of radiopharmaceuticals labeled with these radionuclides.

**Graphical abstract:**

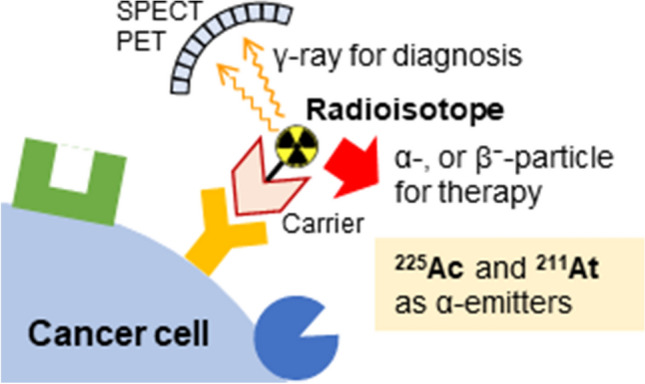

## Introduction

Radiotheranostics is a promising medical technology that uses a set of radioligands incorporating diagnostic or therapeutic radionuclides to achieve both diagnosis and therapy. For instance, by incorporating diagnostic radionuclides into cancer targeting agents, imaging diagnosis can provide information about the presence of targets and the accessibility of the agents. Subsequently, the introduction of therapeutic radionuclides into these imaging probes holds the potential to enable precise radionuclide therapy [1–3]. Among them, targeted alpha therapy (TAT) is a cancer treatment approach that uses tumor-homing agents with α-particle-emitting radionuclides (α-emitters) [4, 5]. The α-particles emitted from the TAT agents exhibit a constrained tissue range, usually affecting only a few number of cells (50–100 μm), enabling the specific irradiation of the target cancer cells. Moreover, α-particles possess a high linear energy transfer (LET) ranging from 50 to 230 keV μm^−1^ [6], enabling them highly effective in inducing cell death, primarily through the induction of double-strand breaks in DNA [7]. Therefore, TAT is expected to be a precise therapy that can regress cancer cells while protecting healthy tissues. TAT is expected to revolutionize cancer treatment, by bringing a novel perspective to late-stage cancer, as treatment options are limited, and contributing to major advances in the field of cancer treatment [8].

**Fig. 1:**
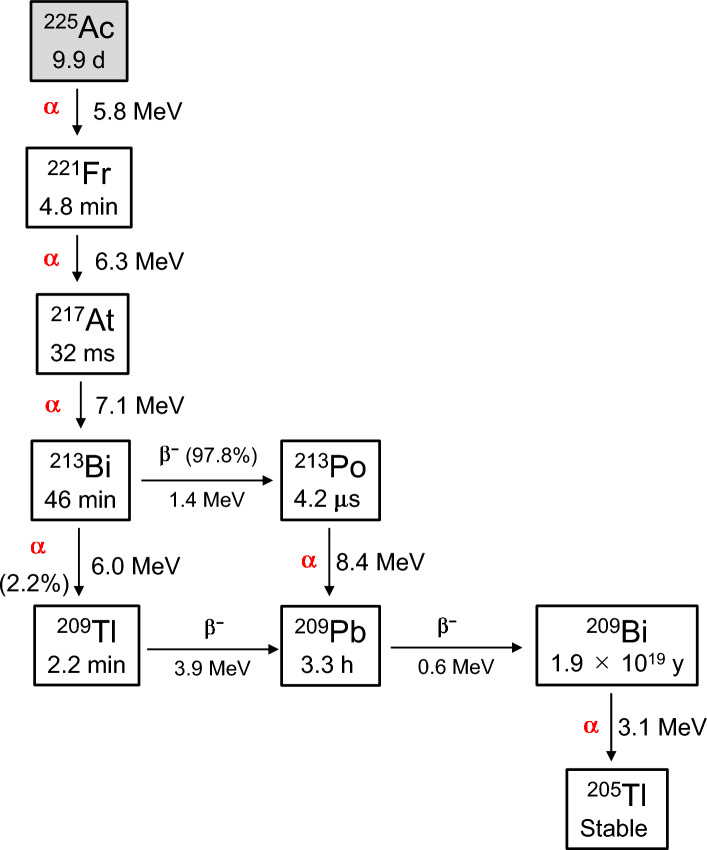
Decay scheme of ^225^Ac

**Fig. 2:**
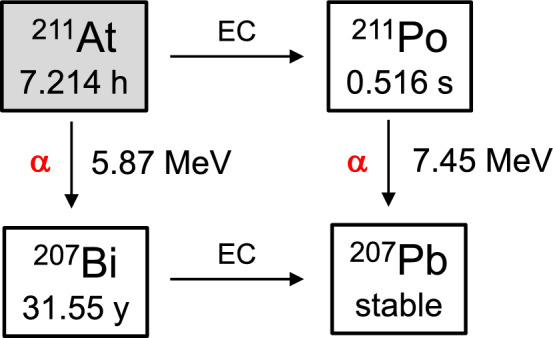
Decay scheme of ^211^At

Several useful α-emitters, including ^223^Ra, ^225^Ac, and ^211^At, are currently used in clinical treatment modalities and clinical trials [9]. ^223^Ra has a half-life of 11.4 days, and its ionic form, [^223^Ra]Ra^2+^, is clinically employed in treating bone metastatic prostate cancer as a commercially available radiopharmaceutical named Xofigo [10]. ^223^Ra has a reasonably long half-life and is anticipated to be a valuable nuclide for TAT. Nevertheless, developing an appropriate stable chelator of ^223^Ra for clinical applications is presently a challenging obstacle, impeding progress in the development of radiotracers for various targets. ^225^Ac has multiple α-particles with high energy (5.8–7.1 MeV) and sufficient half-life (t_1/2_ = 9.9 days) for high therapeutic efficacy (Fig. 1) [11]. Furthermore, it can establish stable complexes by binding to ligands like 1,4,7,10-tetraazacyclododecane-1,4,7,10-tetra-acetic acid (DOTA), enabling its use as versatile bifunctional agents within any cancer-targeted molecules [12]. Hence, ^225^Ac is recognized as one of the most effective α-emitters for cancer therapy. The release of radioactivity from target tumor tissues owing to the desorption of the daughter nuclides of ^225^Ac from the chelator is a problem that needs to be addressed. In addition, the restricted availability of ^229^Th, which serves as the primary source of ^225^Ac, hinders the global distribution of this radionuclide. Recently, ^211^At has also been considered as a promising α-emitter for TAT [13]. The relatively short half-life of ^211^At (*t*_1/2_ = 7.2 h) gives rise to various issues, including the challenges of guaranteeing an ample supply of therapeutic doses and facilitating the distribution of ^211^At from manufacturing facilities to medical institutions where it is employed. Conversely, ^211^At possesses distinct advantages over other α-emitters with longer half-lives, like ^225^Ac.^211^At is produced by the nuclear reaction of ^209^Bi(α, 2n)^211^At using a cyclotron from ^209^Bi, which is relatively easy to obtain. ^211^At decays with 5.87MeV of α-emission to transform into ^207^Bi, which subsequently decays via electron capture (EC) into stable ^207^Pb (Fig. 2). In the second branched decay, ^211^At can also undergo EC decay to form ^211^Pb, followed by the emission of α-particle (7.45 MeV) to produce ^207^Pb. In other words, ^211^At emits 100% α-particles in decay, and unlike the decay of ^225^Ac, long-lived α-particle-emitting daughter nuclides are not produced [14]. Similar to its cognate halogen atoms, ^123/131^I, ^211^At forms biologically stable molecules that covalently binds to benzene rings and neopentyl groups [15, 16]. It is expected to serve as a versatile bifunctional molecules that binds to cancer-targeting molecules, such as ^225^Ac-labeled agents, to develop diverse TAT agents. Another major advantage is the ease of imaging the biodistribution of ^211^At-labeled compounds by detecting ^211^Po-derived X-rays with a gamma camera or single photon emission computed tomography (SPECT) [17].

In this context, a recent surge has been observed in research focused on the development and clinical applications of new drugs labeled with ^225^Ac and ^211^At. This review focuses on the recent advances in radiopharmaceuticals labeled with ^211^At and ^225^Ac and offers a comprehensive overview of their synthesis, biological evaluation, and clinical applications.

## Radiolabeled compounds for thyroid cancers

Thyroid cancer therapy is based on surgery followed by radioiodine therapy. Radioiodine treatment with radioiodine diagnosis was first conducted by Dr. Hertz in 1942 [18–20], marking the beginning of radiotheranostics. Radioiodine diagnosis and treatment are based on iodine uptake into differentiated thyroid cancer cells by the sodium/iodide symporter (NIS), and this theranostic strategy is applicable for NIS-expressing cancers including metastatic regions. Radioiodine has been used to treat thyroid diseases for more than 80 years. However, some patients with multiple metastases are refractory to repetitive radioiodine (^131^I) treatment despite sufficient iodine uptake in targeted regions [21, 22]. A more effective strategy is required to treat radioactivity-refractory cancer in such cases.

^211^At, a halogen element with chemical properties similar to those of iodine, has been gained attention as an α-emitter. [^211^At]Astatide also accumulates in cancer cells mediated by NIS [23, 24].These characteristics are similar to those of [^131^I]iodide, suggesting that [^211^At]astatide is a possible alternative radionuclide to [^131^I]iodide in NIS-based endoradiotherapy. A toxicity study demonstrated no severe side effects in normal mice intravenously administered with [^211^At]NaAt solutions up to 50 MBq/kg [25]. In addition, the [^211^At]NaAt induced more DNA double-strand breaks and decreased colony formation than [^131^I]NaI and a stronger tumor-growth suppression was observed in mice injected with 0.4 and 0.8 MBq of [^211^At]NaAt than those injected with 1.0 MBq of [^131^I]NaI [26]. Furthermore, therapeutic experiments using NIS-expressing tumor-bearing mice demonstrated complete primary tumor eradication with no recurrence during 1-year follow-up [27]. The major features of ^211^At are a potent therapeutic effect and an extremely short range that reduces radiation exposure to surrounding people, enabling outpatient treatment without requiring admission to a dedicated hospital room. Therefore, Phase I trials are currently underway in Japan. [^211^At]NaAt is being investigated in patients with differentiated thyroid cancer at Osaka University Hospital to establish the recommended dose for Phase II trials (NCT05275946).

## Radiolabeled compounds for norepinephrine (NE) transporter-expressing cancers

**Fig. 3:**
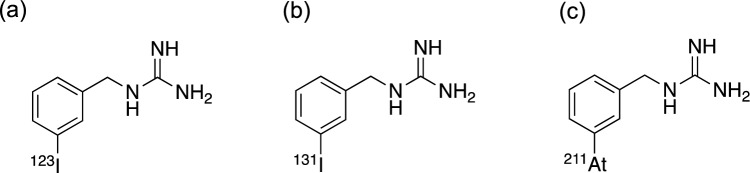
Chemical structures of [^123^I]MIBG (**a**), [^131^I]MIBG (**b**), and [^211^At]MABG (**c**)

Neuroblastoma is a pediatric cancer originating from the sympathetic nervous system, often characterized by metastasis and recurrence, and is often inoperable in many instances [28, 29]. Pheochromocytomas and paragangliomas are rare neuroendocrine tumors (NETs) associated with a relatively high incidence of local invasion or metastasis, rendering some cases unsuitable for surgical intervention [30, 31]. Most of these tumors express high levels of NE transporters [28, 32]. Because *m*-[^123^I]iodobenzylguanidine ([^123^I]MIBG) (Fig. 3a) is a substrate for the NE transporter, SPECT imaging with this radioligand has been used to diagnose these tumors such as neuroblastomas [33]. The *m*-[^131^I]iodobenzylguanidine ([^131^I]MIBG) (Fig. 3b), wherein the β^−^-emitter ^131^I replaces ^123^I, has been clinically utilized as an effective therapeutic radioligand for tumors expressing the NE transporter. Response rates of over 30% have been observed when administered as a single agent [34, 35]. Nevertheless, its effect is frequently short-lived because the β^−^-particles from [^131^I]MIBG may not be optimal for effectively eradicating isolated cells or small cell clusters due to their extended path lengths [36, 37]. Hence, *m*-[^211^At]astatobenzylguanidine ([^211^At]MABG), where the *meta*-position ^131^I of [^131^I]MIBG is substituted with ^211^At (Fig. 3c), α-emitter capable of focusing high energy within a more confined area, gained attention. [^211^At]MABG demonstrated the physicochemical properties similar to those of [^131^I]MIBG and specific uptake by neuroblastoma cells in vitro [38]. [^211^At]MABG showed a similar disposition to [^131^I]MIBG in SK-N-SH tumor-bearing mice but with higher accumulation in the tumor and heart [39]. Accordingly, [^211^At]MABG was anticipated to pave the way for a novel TAT that could surpass existing treatments for NE transporter-expressing tumors. [^211^At]MABG showed notably higher cytotoxicity in the non-exposed group than in spheroids consisting of SK-N-BE(2c) in neuroblastoma from 0.48 kBq/mL [40]. The maximum tolerated dose of [^211^At]MABG ranged from 51.8 to 66.7 MBq/kg in a mouse model of disseminated neuroblastoma transplanted with cells that overexpress the NE transporter. The results indicated that a single dose (66.7 MBq) or four divided doses (16.6 MBq) resulted in notable tumor regression effects and extended survival [41]. [^211^At]MABG has also shown remarkable therapeutic efficacy in treating malignant pheochromatoma. The administration of [^211^At]MABG (0.56 MBq) to rat pheochromatoma PC12 tumor-bearing mice resulted in a notable tumor regression effect, with tumors being 53 times smaller after 21 days than those in the control group (relative tumor volumes of 509% and 9.6% when compared to control, respectively) [42]. Analysis of mRNA expression in response to [^211^At]MABG indicated that change in the p53-p21-dependent cell cycle checkpoint notably inhibits the growth of PC12 cells [43]. Evaluation of the acute radiation-related toxicity of [^211^At]MABG in ICR mice revealed that a maximum tolerated dose of 3.3 MBq. Despite the high absorbed doses in numerous organs, such as the thyroid, heart, stomach, and adrenal glands, no unexpected severe toxic effects were observed in the mice [44]. Fukushima Medical University Hospital has commenced a phase I dose-escalation study of [^211^At]MABG in patients diagnosed with malignant pheochromocytoma or paraganglioma (jRCT2021220012) [13]. On the other hand, the uptake of [^211^At]MABG by the non-target organic cation transporter 3 poses a risk of potential side effects in normal tissues, and, therefore, warrants careful consideration in the treatment process [45]. The combination of histone deacetylase inhibitors such as Vorinostat and [^211^At]MABG exhibits a synergistic neuroanticancer effect on neuroblastoma. This effect may be attributed to reduced expression of DNA damage repair proteins and increased expression of NE transporter proteins [46, 47]. Additional basic and clinical studies of [^211^At]MABG are anticipated in the future, including investigations to confirm whether combination therapy with other agents can enhance its therapeutic effectiveness.

## Radiolabeled octreotide analogs with high affinity for somatostatin receptors

**Fig. 4:**
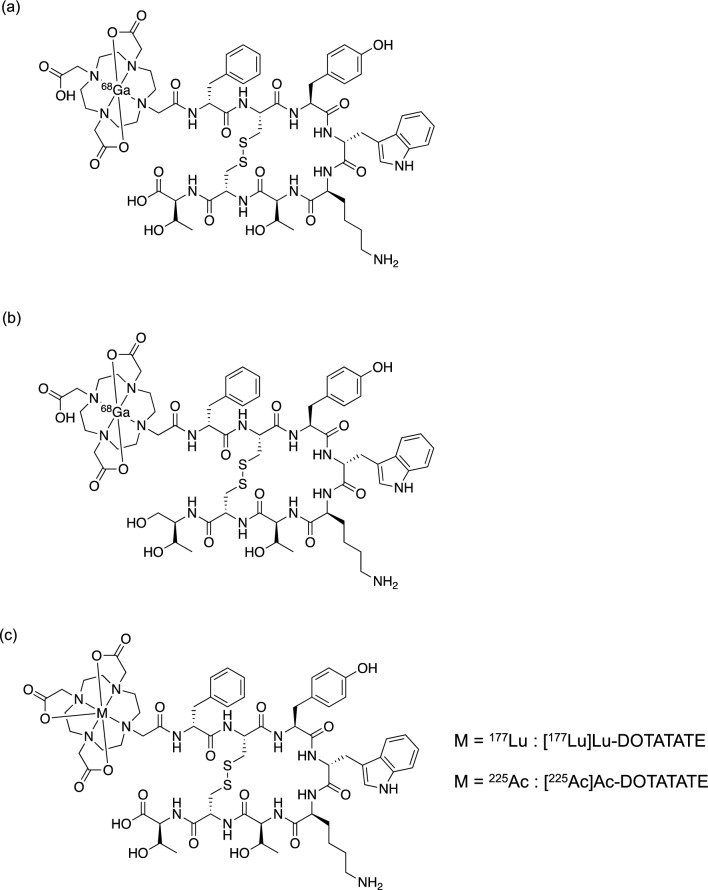

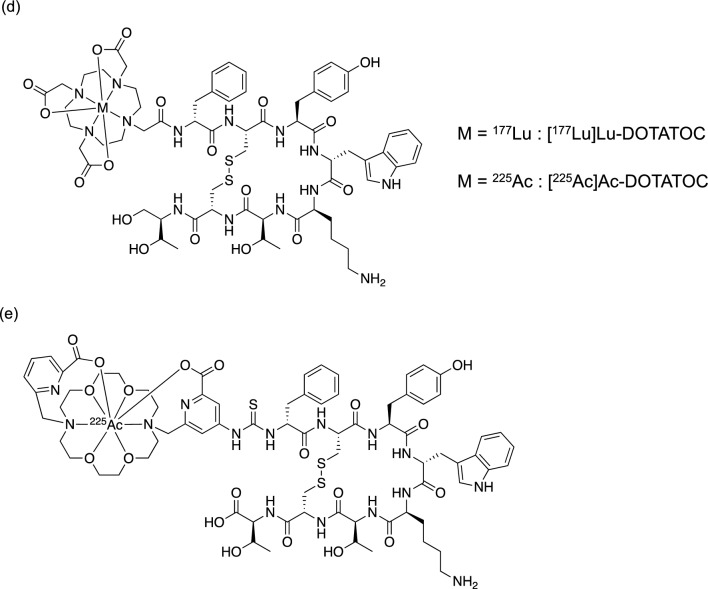
Chemical structures of [^68^Ga]Ga-DOTATATE (**a**), [^68^Ga]Ga-DOTATOC (**b**), DOTATATE derivatives labeled with ^177^Lu or ^225^Ac (**c**), DOTATOC derivatives labeled with ^177^Lu or ^225^Ac (**d**), and [^225^Ac]Ac-MACROPATATE (**e**)

NETs are neoplasms arising from endocrine cells primarily found in the gastrointestinal tract, pancreas, lungs, and other tissues [48]. NETs typically demonstrate a highly differentiated, low-proliferative character and often require surgical intervention for a complete cure [49]. However, in some cases, they may be unresectable during detection and chemotherapy tends to be less effective. Somatostatin receptors (SSTRs), particularly SSTR2, are highly expressed in NETs. Consequently, ^68^Ga-labeled octreotide derivatives of cyclic peptides, including [^68^Ga]Ga-DOTATATE (Fig. 4a) and [^68^Ga]Ga-DOTATOC (Fig. 4b), have been employed for positron emission tomography (PET) diagnosis of tumors expressing SSTRs and for providing prognostic information [50]. For radionuclide therapy, the clinical application of [^177^Lu]Lu-DOTATATE (Fig. 4c) radiolabeled with a β^−^-emitter ^177^Lu with specific affinity for SSTR2, has proven efficacious in the treatment of metastatic and unresectable NETs [51]. However, certain tumors demonstrate resistance or recurrence when subjected to this therapeutic approach [52]. The lower LET of β^−^-particles from ^177^Lu (~ 0.2 keV/μm) compared to α-particles is associated with their primary mechanism of inducing single-strand DNA breaks, which may explain their limited therapeutic effectiveness. Therefore, in anticipation of the efficacy of NETs with TAT, fundamental and clinical studies on the therapeutic effects on NETs of octreotide derivatives labeled with ^225^Ac, which can form stable complexes with DOTA as well as ^177^Lu, were subsequently conducted [8, 53].

**Fig. 5:**
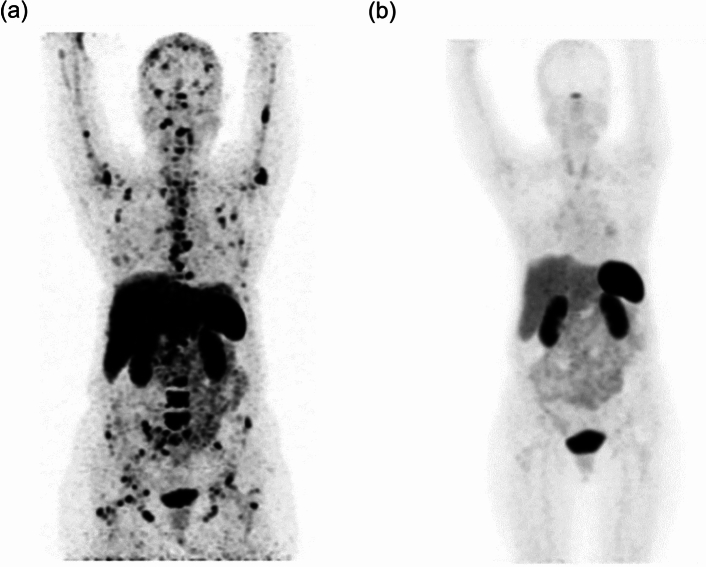
A 54-year-old woman with rectal NET received combination therapy of [^177^Lu]Lu-DOTATATE and capecitabine. Initial [^68^Ga]Ga-DOTANOC PET/CT revealed widespread skeletal metastases (a). After two cycles of [^225^Ac]Ac-DOTATATE, follow-up scan indicated partial morphological and molecular response. Reproduced with some modifications from *Eur J Nucl Med Mol Imaging*, **47**, 934–946 (2020), with permission [64]

Cyclic peptides DOTATOC and DOTATATE, known for their high affinity for SSTR2 and labeled with radiometals (^68^Ga for diagnostic purposes and ^90^Y and ^177^Lu for therapy), are already employed in clinical practice [54]. Accordingly, the initial preclinical investigations focused on DOTA peptides labeled with ^225^Ac (Fig. 4c, d). [^225^Ac]Ac-DOTATOC (12–20 kBq) suppressed the growth of NETs inoculated in mice more effectively than [^177^Lu]Lu-DOTATOC (450–1000 kBq), and no toxicity was observed up to 20 kBq [55]. A single administration of [^225^Ac]Ac-DOTATATE (144–148 kBq) resulted in a remarkable tumor growth delay and extended the time to the experimental endpoint in SSTR2-positive lung cancer cell-transplanted mice compared to that of the control group [56]. Both [^225^Ac]Ac-DOTATOC and [^225^Ac]Ac-DOTATATE exhibited nephrotoxicity at high doses (30 and 111 kBq, respectively), which was attributed to their substantial renal accumulation. [^225^Ac]Ac-MACROPATATE (Fig. 4e) showed better serum stability with a chelator different from that of [^225^Ac]Ac-DOTATATE. However, its antitumor effect was lower than that of [^225^Ac]Ac-DOTATATE, its accumulation in the liver and kidney was higher, and its superiority over existing radioligands has not been verified [57]. A preclinical study on lung cancer-bearing mice treated with ^211^At-labeled octreotide ([^211^At]SAB-Oct) has also been reported. Significant tumor regression was observed after 370 kBq administration compared with that of control group. A total of 1110 kBq administered in triplicate showed no noticeable toxicity or activation of the antitumor immune response [58]. Due to their unchanged binding and slow dissociation rates, SSTR antagonists are promising ligands for TAT. [^177^Lu]Lu-DOTA-LM3 and [^225^Ac]Ac-DOTA-LM3 have shown good tumor regression in clinical studies [59, 60]. Recently, the SSTR antagonist [^225^Ac]Ac-DOTA-JR11 was developed, which showed good tumor accumulation but relatively high uptake in the kidney, liver, and bone [61]. Among the ^225^Ac-labeled octreotide analogs, only [^225^Ac]Ac-DOTATOC and [^225^Ac]Ac-DOTATATE have been studied clinically, owing to the overwhelming abundance of clinical data on these scaffolds. A single dose (9.8 MBq) of [^225^Ac]Ac-DOTATOC has been reported to achieve partial remission without side effects in patients with pancreatic NET and liver metastases refractory to treatment with [^177^Lu]Lu-DOTATATE [62]. A 5-year long-term follow-up study of [^225^Ac]Ac-DOTATOC administration was conducted in patients for whom other treatments were not viable. Nephrotoxicity was observed though not dependent on the amount of radioactivity, and no relationship with TAT was known. Although dose-dependent hematologic toxicity (over 40 MBq of a single dose or over 20 MBq of repeated doses) was observed, it was concluded that with appropriate dose control, [^225^Ac]Ac-DOTATOC-based TAT could be a safe and effective treatment [63]. After [^177^Lu]Lu-DOTATATE treatment, 32 metastatic NET patients received [^225^Ac]Ac-DOTATATE (100 kBq/kg) every eight weeks (Fig. 5). This led to partial remission and stability, with no progression or death in the 8-month follow-up [64]. Recently, [^225^Ac]Ac-DOTATATE therapy improved overall survival of 91 patients with SSTR-expressing NETs. Treatment-related toxicity was minimal, suggesting that overall survival could be improved even in patients refractory to previous [^177^Lu]Lu-DOTATATE therapy [65]. Several other case reports have also highlighted the clinical advantages of [^225^Ac]Ac-DOTATATE, including complete remission in multiple patients [66–69]. Although persistent concerns regarding nephrotoxicity are likely to drive the development of new ^225^Ac-labeled SSTR-targeted agents, TAT with [^225^Ac]Ac-DOTATATE has great potential as a potent treatment for NETs in clinical practice.

## Bone-seeking radionuclides or radiolabeled bone-seeking compounds

Many bone-seeking agents with β^−^-emitter for palliation of bone metastases, such as [^89^Sr]SrCl_2_ and [^153^Sm]Sm-EDTMP, have been developed for a long time [70, 71]. However, bone-seeking radiopharmaceuticals with β^−^-emitters do not prolong the overall survival in patients. Meanwhile, [^223^Ra]RaCl_2_ significantly prolonged the overall survival of castration-resistant prostate cancer patients with bone metastases in a phase III study [72]. Following the results of the phase III study, [^223^Ra]RaCl_2_ was approved by the U.S. Food and Drug Administration (FDA) as the first therapeutic radiopharmaceutical with an α-emitter. Although [^223^Ra]RaCl_2_ and the bone scintigraphy agents do not have precisely equivalent pharmacokinetics, they accumulate in bones with high osteoblastic activity, such as bone metastases. The lesion uptake of [^223^Ra]RaCl_2_ was reported to significantly correlate with that of [^99m^Tc]Tc-MDP [73]. Therefore, bone scintigraphy agents, such as [^99m^Tc]Tc-MDP, are used as companion diagnostic imaging agents for [^223^Ra]RaCl_2_.

**Fig. 6:**
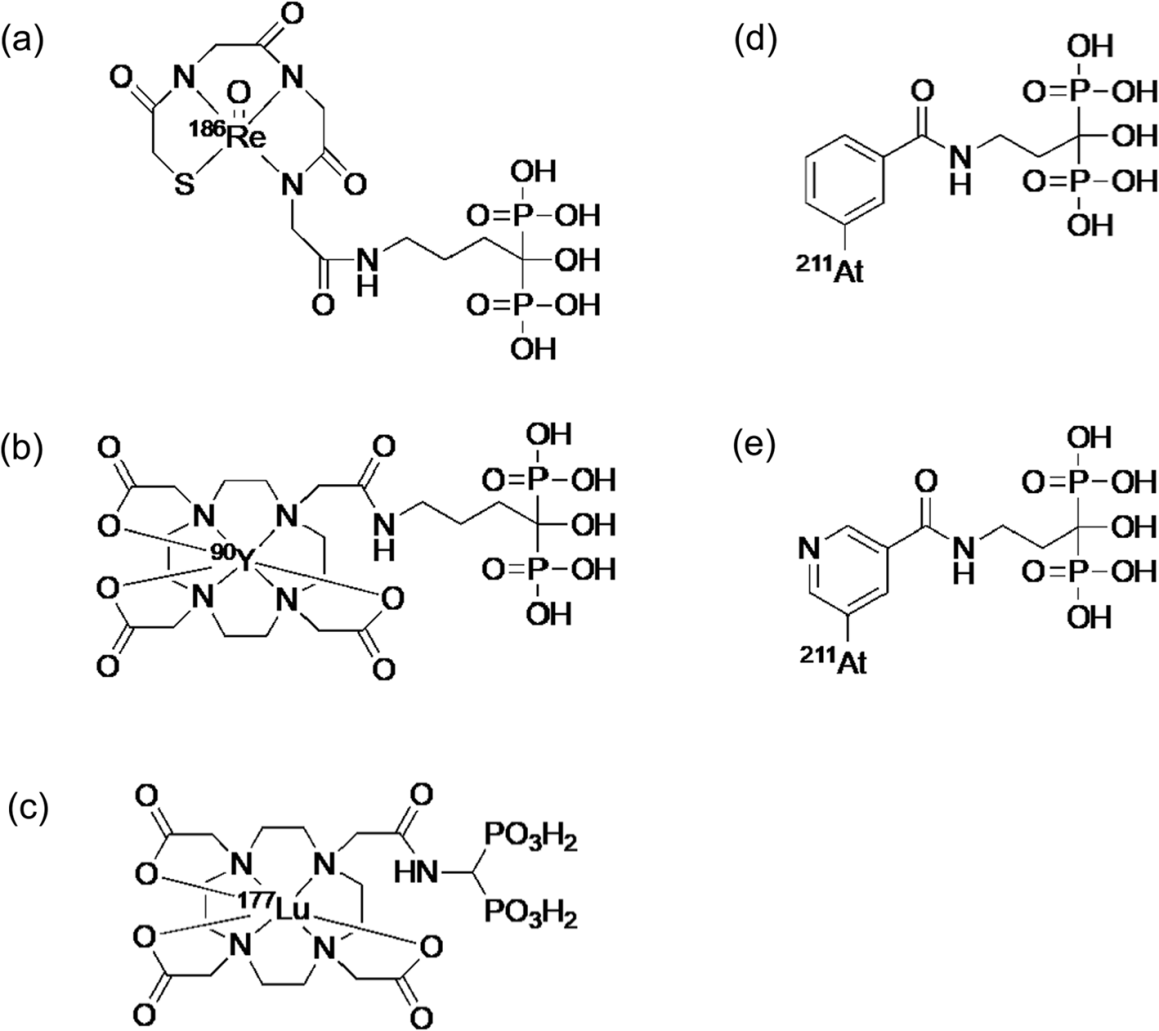
Structures of [^186^Re]Re-MAG3-HBP (**a**), [^90^Y]Y-DOTA-HBP (**b**), [^177^Lu]Lu-BPAMD (**c**), [^211^At]ABPB (**d**), and [^211^At]APPB (**e**)

In basic research, complexes with β^−^-emitters, such as [^186^Re]Re-MAG3, [^90^Y]Y-DOTA, and [^177^Lu]Lu-DOTA, conjugated bisphosphonate compounds, which are carriers to bone lesions, were developed for the palliation of bone metastases (Fig. 6a–c) [74–76]. These compounds showed high uptake in bone and low uptake in non-target tissues, indicating that the drug design concept is useful for bone-seeking radiopharmaceuticals. Moreover, the replacement of radionuclides for therapy to ones for imaging could adopt ideal radiotheranostics because diagnostic and therapeutic radiopharmaceuticals could show equivalent pharmacokinetics [77–79].

Using a similar concept, ^211^At introduced bisphosphonate derivatives, 3-[^211^At]astato-benzamide-*N*-3-hydroxypropylidene-3,3-bisphosphonate ([^211^At]ABPB) and 5-[^211^At]astatopyridine-3-amide-*N*-3-hydroxypropylidene-3,3-bisphosphonate ([^211^At]APPB), were reported (Fig. 6d, e) [80]. These compounds showed high in vivo stability, bone uptake, and rapid clearance from blood. The bone uptake and bone-to-tissue ratios were better for [^211^At]ABPB than for [^211^At]APPB. In radiotheranostics, ^211^At can be replaced with ^123/124^I for SPECT or PET imaging.

## Radiolabeled RGD peptides with high affinity for α_v_β_3_ integrin-expressing cancers

RGD peptides contain arginine–glycine–aspartic acid (RGD) sequence. RGD peptides have a high affinity for α_v_β_3_ integrin, which is a heterodimeric transmembrane receptor for cell adhesion molecule [81]. α_v_β_3_ integrin, one of the integrin subtypes, regulates angiogenesis and is related to tumor development [82]. As the α_v_β_3_ integrin is highly expressed on endothelial cells in neovascularity and some types of cancer cells, RGD peptides have been used as carriers to cancer tissue [83, 84].

Radiolabeled RGD peptides have been enthusiastically developed for cancer imaging and therapy in nuclear medicine [85–87]. Utilization of the RGD tripeptide had been hindered by its short half-life in the blood and insufficient affinity. To overcome the limitation, structural modifications involving the incorporating an additional two amino acids, utilizing D-amino acid residues, and cyclizing the peptides have been implemented to improve the affinity for α_v_β_3_ integrin and its bioavailability. Notably, c(RGDfK) and c(RGDyK) have emerged as fundamental constructs for developing radiolabeled RGD peptides [88]. Furthermore, multimeric RGD peptides, such as dimer and tetramer, have been investigated to enhance affinity for α_v_β_3_ integrin [89]. Radiolabeled RGD peptides were explored for imaging purposes to determine α_v_β_3_ integrin expression. Subsequently, these investigations were extended to the field of targeted radionuclide therapy.

**Fig. 7:**
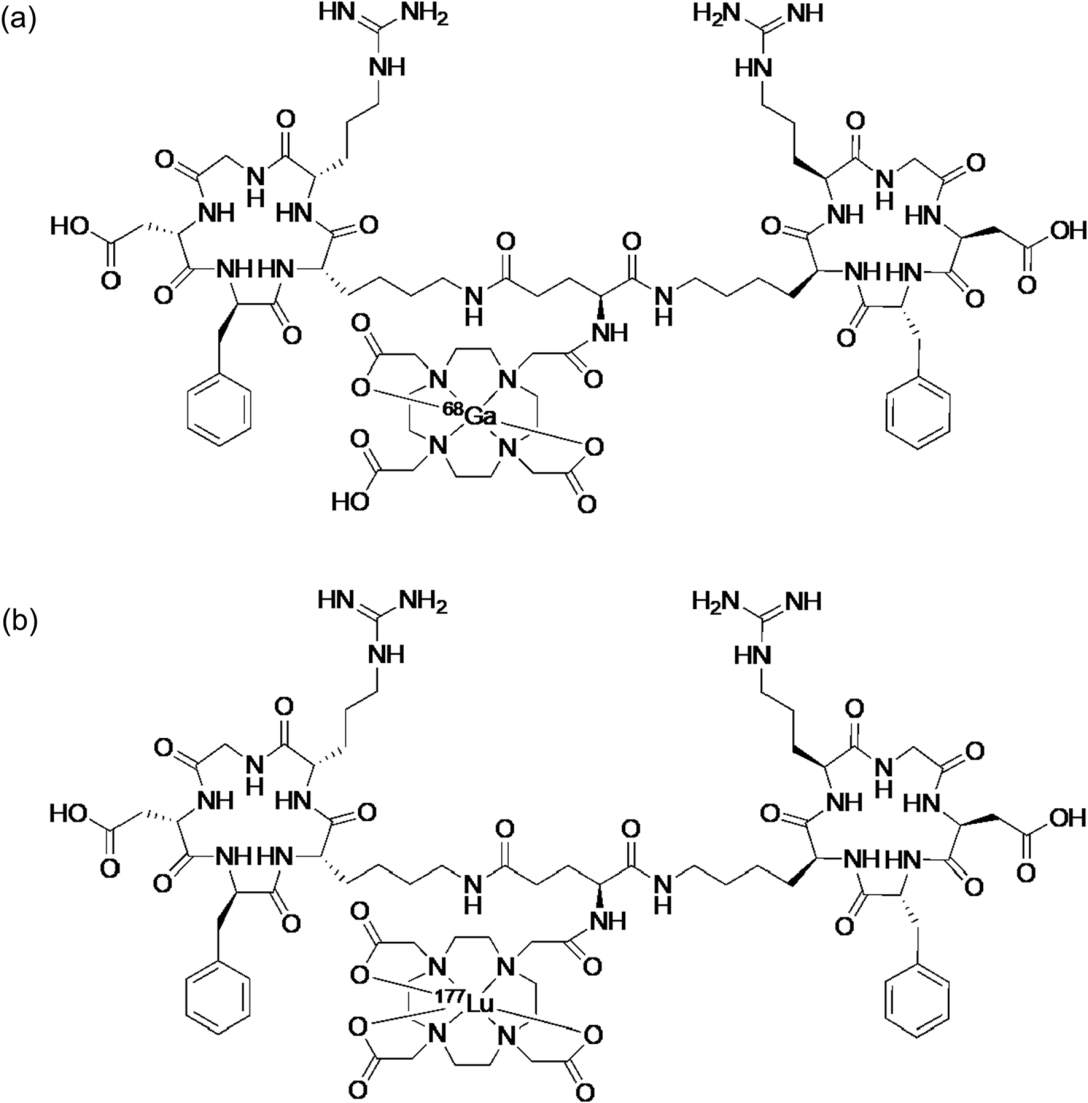
Structures of [^68^Ga]Ga-DOTA-E[c(RGDfK)]_2_ and [^177^Lu]Lu-DOTA-E[c(RGDfK)]_2_

**Fig. 8:**
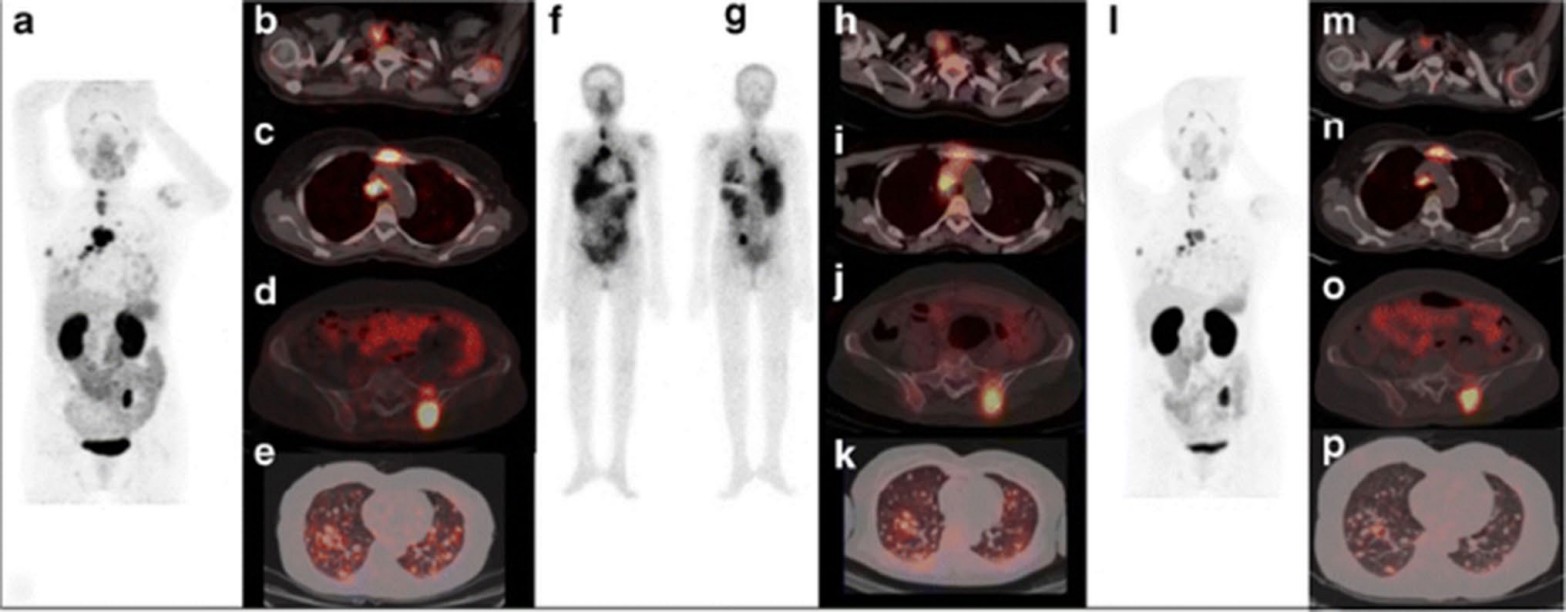
The maximum intensity projection (MIP) image of [^68^Ga]Ga-DOTA-E[c(RGDfK)]_2_ PET/CT for pretreatment assessment (**a**) and transaxial fused PET/CT images showed increased tracer uptake in the thyroid remnant [maximum standardized uptake value (SUVmax) = 4.7] with cervical lymph nodes (**b**), mediastinal lymph node (**c**; SUVmax = 8.4), lytic skeletal lesions with soft tissue component in the sternum (**c**; SUVmax = 7.8) and left iliac bone (**d**; SUVmax = 8.4) and multiple lung nodules (**e**). The patient received 5.5 GBq of [^177^Lu]Lu-DOTA-E[c(RGDfK)]_2_ with post-therapy whole-body images in anterior (**f**) and posterior (**g**) views revealing the overall distribution of [^177^Lu]Lu-DOTA-E[c(RGDfK)]_2_^177^Lu-DOTA-RGD_2_ and transaxial fused SPECT/CT images (**h**–**k**) showing tracer uptake at sites corresponding to [^68^Ga]Ga-DOTA-E[c(RGDfK)]_2_-avid lesions. Post-therapy follow-up [^68^Ga]Ga-DOTA-E[c(RGDfK)]_2_ PET/CT MIP image (**l**) and transaxial fused PET/CT images showed tracer uptake in the thyroid remnant (SUVmax = 3.0 vs 4.7) with cervical lymph nodes (**m**), mediastinal lymph node (**n**; SUVmax = 7.7 vs 8.4), lytic skeletal lesions with significant reduction in soft tissue component in the sternum (*n*; SUVmax = 6.6 vs 7.8) and left iliac bone (**o**; SUVmax 8.1 vs 8.4) and multiple lung nodules (**p**), suggesting response to therapy. This research was originally published in *EJNMMI *[90]

The first report on radiotheranostics application with RGD peptide in a patient with papillary thyroid carcinoma was published in 2018 [90]. This report described a combination of [^68^Ga]Ga-DOTA-E[c(RGDfK)]_2_ for PET imaging and [^177^Lu]Lu-DOTA-E[c(RGDfK)]_2_ for therapy (Figs. 7 and 8). [^177^Lu]Lu-DOTA-E[c(RGDfK)]_2_ accumulated at sites corresponding to the [^68^Ga]Ga-DOTA-E[c(RGDfK)]_2_-avid lesions. [^68^Ga]Ga-DOTA-E[c(RGDfK)]_2_ PET/CT imaging after [^177^Lu]Lu-DOTA-E[c(RGDfK)]_2_ treatment revealed a significant reduction in lesion uptake, indicating a positive therapeutic response. These results suggest a high potential for the radiotheranostics using radiolabeled RGD peptides.

**Fig. 9:**
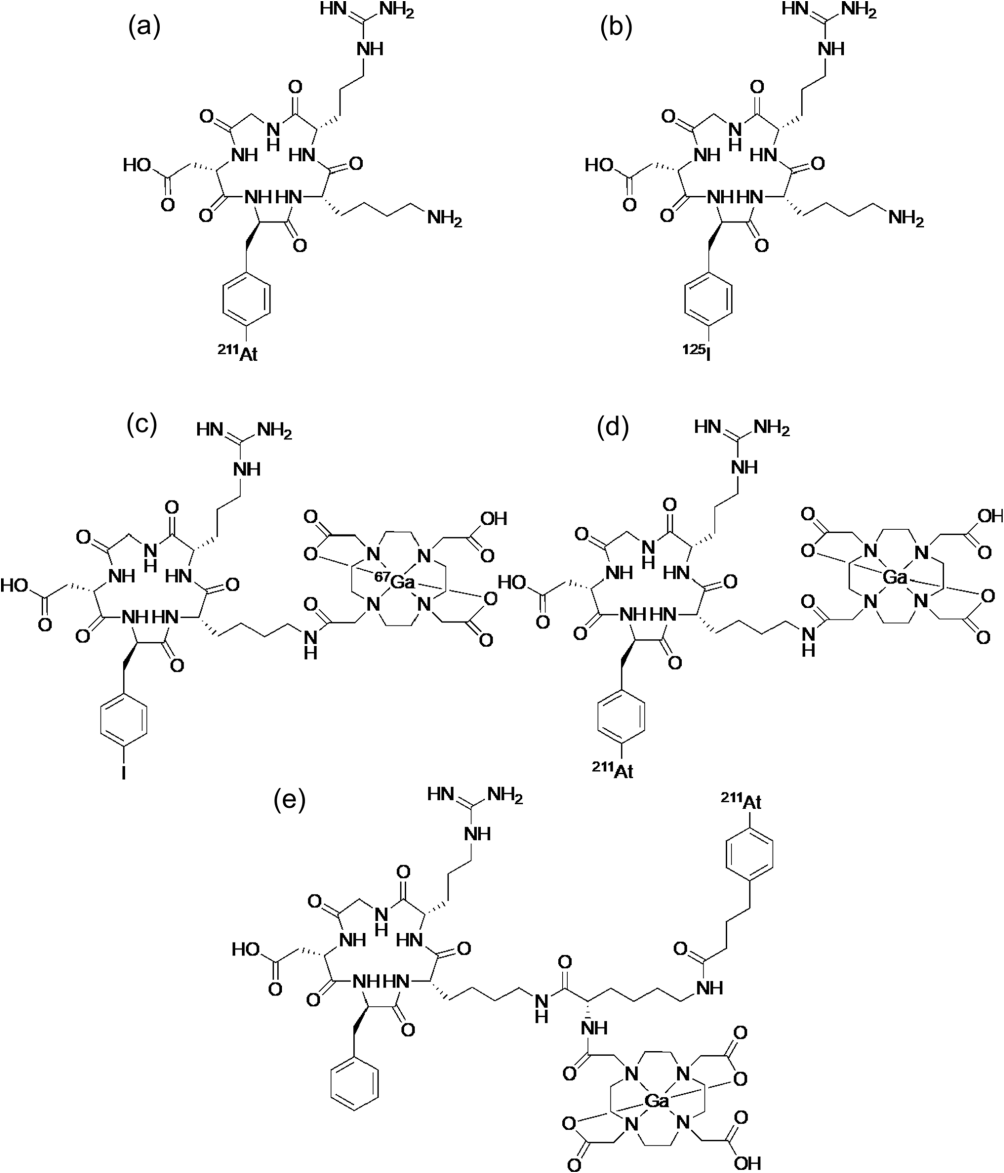
Structures of [^211^At]c[RGDf(4-At)K] (**a**), [^125^I]c[RGDf(4-I)K] (**b**), [^67^Ga]Ga-DOTA-c[RGDf(4-I)K] (**c**), Ga-DOTA-[^211^At]c[RGDf(4-At)K] (**d**), and Ga-DOTA-K([^211^At]APBA)-c(RGDfK) (**e**)

As TAT-targeting α_v_β_3_ integrin, ^211^At-labeled RGD peptide was first reported in 2019 [91]. For ^211^At-labeling of RGD peptides, compound c{RGDf[4-Sn(*n*Bu)_3_]K} was synthesized by introducing a tributyltin group into the d-phenylalanine of c(RGDfK). Labeling reactions were performed to synthesize [^211^At]c[RGDf(4-At)K] and [^125^I]c[RGDf(4-I)K] (Fig. 9a, b). [^211^At]c[RGDf(4-At)K] and [^125^I]c[RGDf(4-I)K] showed high-tumor uptake and an equivalent biodistribution of radioactivity in U87MG tumor-bearing mice, indicating the usefulness of the combination of ^211^At-labeled RGD peptide with the corresponding radioiodine labeled RGD peptide for radiotheranostics.

Radiotheranostics is generally performed by introducing diagnostic and therapeutic radionuclides with similar chemical properties into the same precursor. Therefore, the combinations of radionuclides for radiotheranostics are limited. To overcome the limitation, multiradionuclide radiotheranostics with a combination of [^67^Ga]Ga-DOTA-c[RGDf(4-I)K], in which ^67^Ga is an alternative radionuclide to ^68^Ga, and Ga-DOTA-[^211^At]c[RGDf(4-At)K] were developed by introducing a halogen introduction site and a metal complex in a molecule (Fig. 9c, d) [92]. To increase tumor accumulation and retention of ^211^At-labeled RGD peptide, an albumin-binding moiety (ABM) was introduced (Fig. 9e). Ga-DOTA-K([^211^At]APBA)-c(RGDfK) with ABM delayed blood clearance, increased tumor accumulation compared to compounds without ABM, and showed strong therapeutic effects in tumor-bearing mice [93]. Clinical applications of TAT using RGD peptides are expected in the future.

## Radiolabeled prostate-specific membrane antigen (PSMA) ligands

**Fig. 10:**
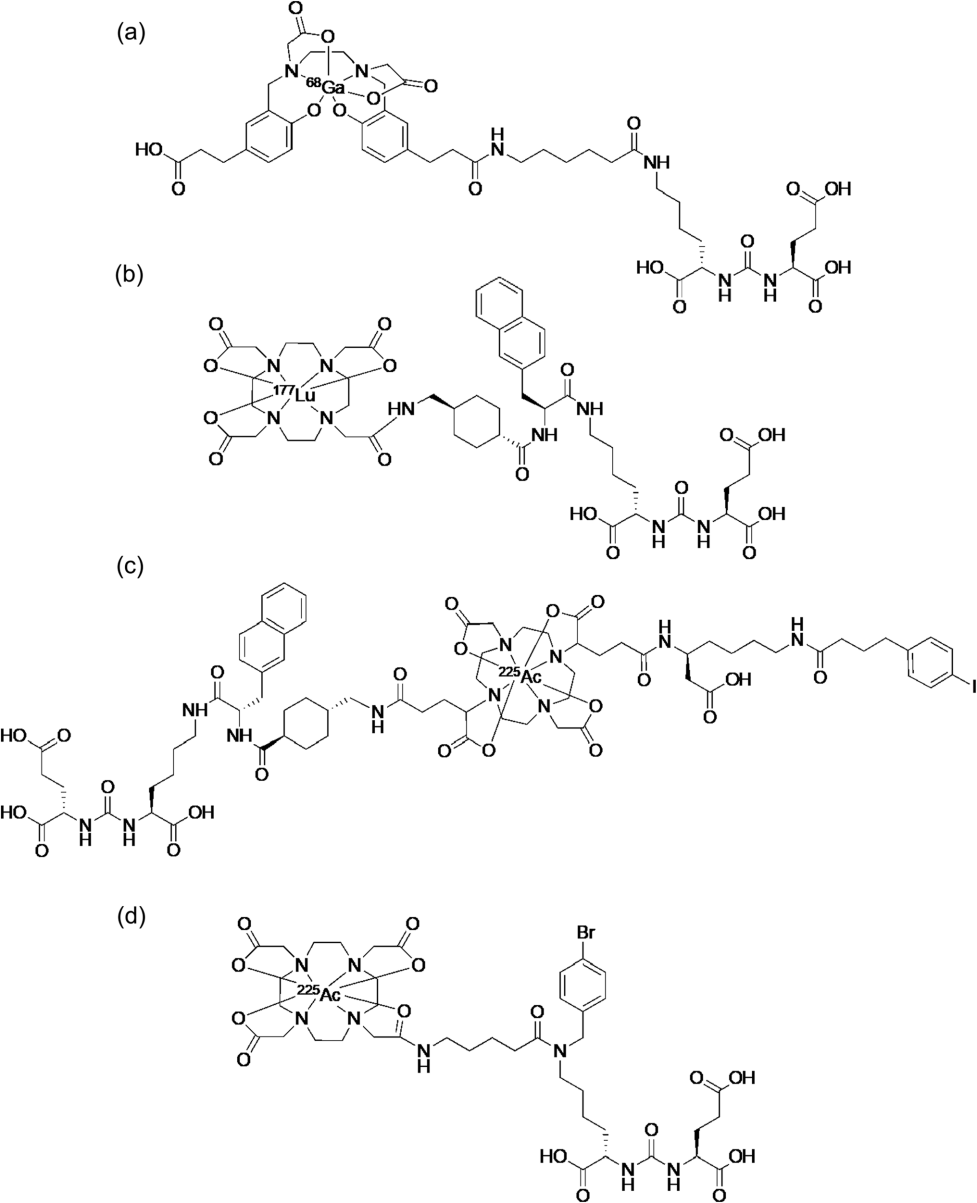
Structures of [^68^Ga]Ga-PSMA-11 (**a**), [^177^Lu]Lu-PSMA-617 (**b**), [^225^Ac]Ac-PNT-DA1 (**c**), and [^225^Ac]Ac-L1 (**d**)

Radiotheranostics combining [^68^Ga]Ga-PSMA-11 and [^177^Lu]Lu-PSMA-617 have been approved and are used in USA and EU [94, 95]; radiotheranostics using prostate-specific membrane antigen (PSMA) ligands in patients with prostate cancer has attracted much attention in recent nuclear medicine (Fig. 10).

PSMA, a cell surface enzyme consisting of 750 amino acids with a molecular weight of 87 kDa, is predominantly expressed in prostate epithelial cells. PSMA is not released into the blood and is overexpressed in prostate cancer, exhibiting a progressive increase in its expression with higher tumor grades [96, 97]. As the PSMA expression level is a significant indicator for predicting disease outcomes in patients with prostate cancer [98], PSMA could be an appropriate target for radiotheranostics.

Almost all radiolabeled PSMA ligands have recently been shown to possess a Glu–urea–Lys pharmacophore. Moreover, PSMA ligands with a lipophilic linker increased the binding affinity for PSMA due to a hydrophobic pocket adjacent to the pharmacophore [99, 100]. In 2012, [^68^Ga]Ga-PSMA-11 (Fig. 10a) was reported to exhibit high PSMA-specific internalization in prostate cancer cells and excellent PET images [101]. Meanwhile, the HBED-CC chelate for ^68^Ga in PSMA-11 does not coordinate with therapeutic radiometals such as ^177^Lu. Subsequently, PSMA-617, a pharmacophore Glu–urea–Lys conjugated DOTA chelator (Fig. 10b) was developed via a lipophilic linker optimized for properties such as length, polarity, size, flexibility, and the presence of aromatic groups [102].

**Fig. 11:**
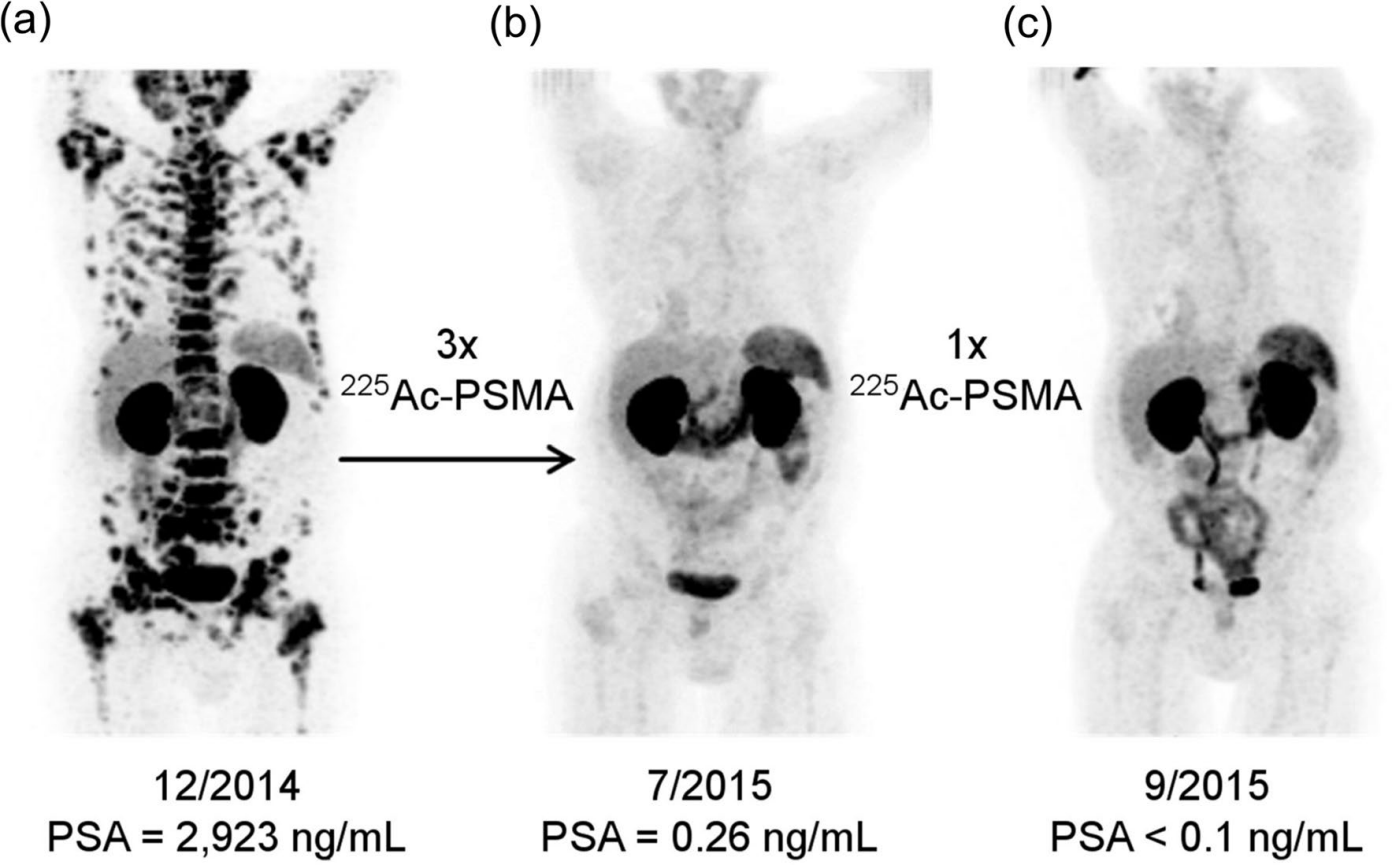
[^68^Ga]Ga-PSMA-11 PET/CT scans of a patient. Pretherapeutic tumor spread (**a**), restaging 2 months after third cycle of [^225^Ac]Ac-PSMA-617 (**b**), and restaging 2 months after one additional consolidation therapy (**c**). This research was originally published in *JNM* [103]

About TAT-targeting PSMA, the surprising therapeutic effects of [^225^Ac]Ac-PSMA-617 were reported in clinical studies in 2016 (Fig. 11) [103]. The initial clinical encounter with [^225^Ac]Ac-PSMA-617 revealed encouraging antitumor efficacy. The duration of response is 10–15 months with complete remission in approximately 10% of patients, while some patients have sustained relapse-free survival [104]. In basic research, superior ^111^In/^225^Ac-labeled compounds targeting PSMA have been developed for cancer radiotheranostics. The pharmacokinetics of the PSMA ligand were improved by introducing ABM into PSMA ligand with a lipophilic linker. The novel ^225^Ac-labeled PSMA ligand, [^225^Ac]Ac-PNT-DA1 (Fig. 10c), showed superior antitumor effects compared to [^225^Ac]Ac-PSMA-617 [105]. Another research group developed an alternative ^225^Ac-labeled PSMA ligand, ^225^Ac-L1, based on a series of ^177^Lu-labeled PSMA with reduced off-target toxicity using Glu–urea–Lys as the targeting moiety (Fig. 10d) [106]. ^225^Ac-L1 showed high uptake in PSMA + PC3 PIP tumors, rapid clearance from the blood and kidneys, and low uptake in other non-target tissues. Moreover, ^225^Ac-L1 inhibited PSMA-specific tumor growth without causing off-target toxicity.

**Fig. 12:**
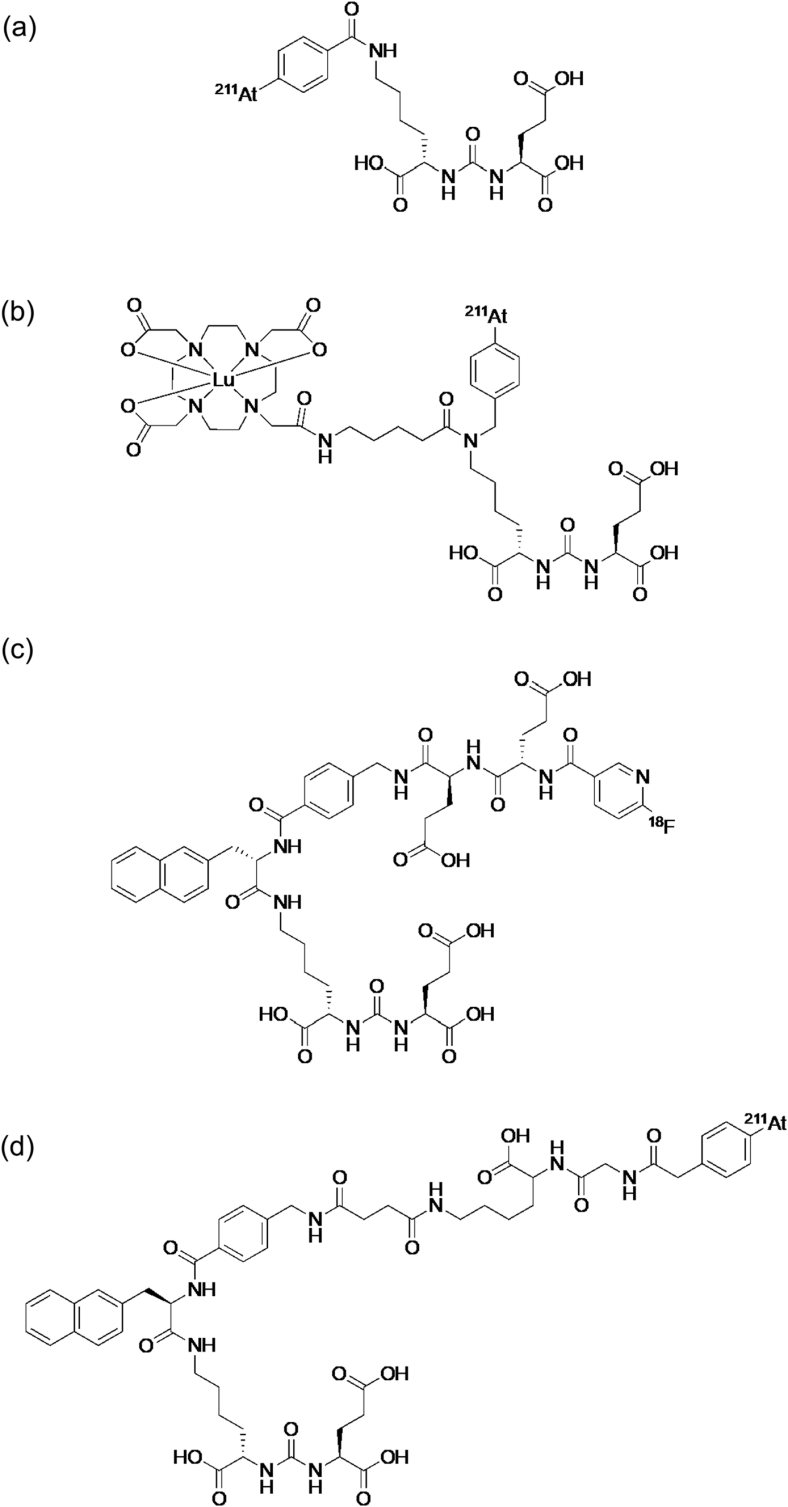

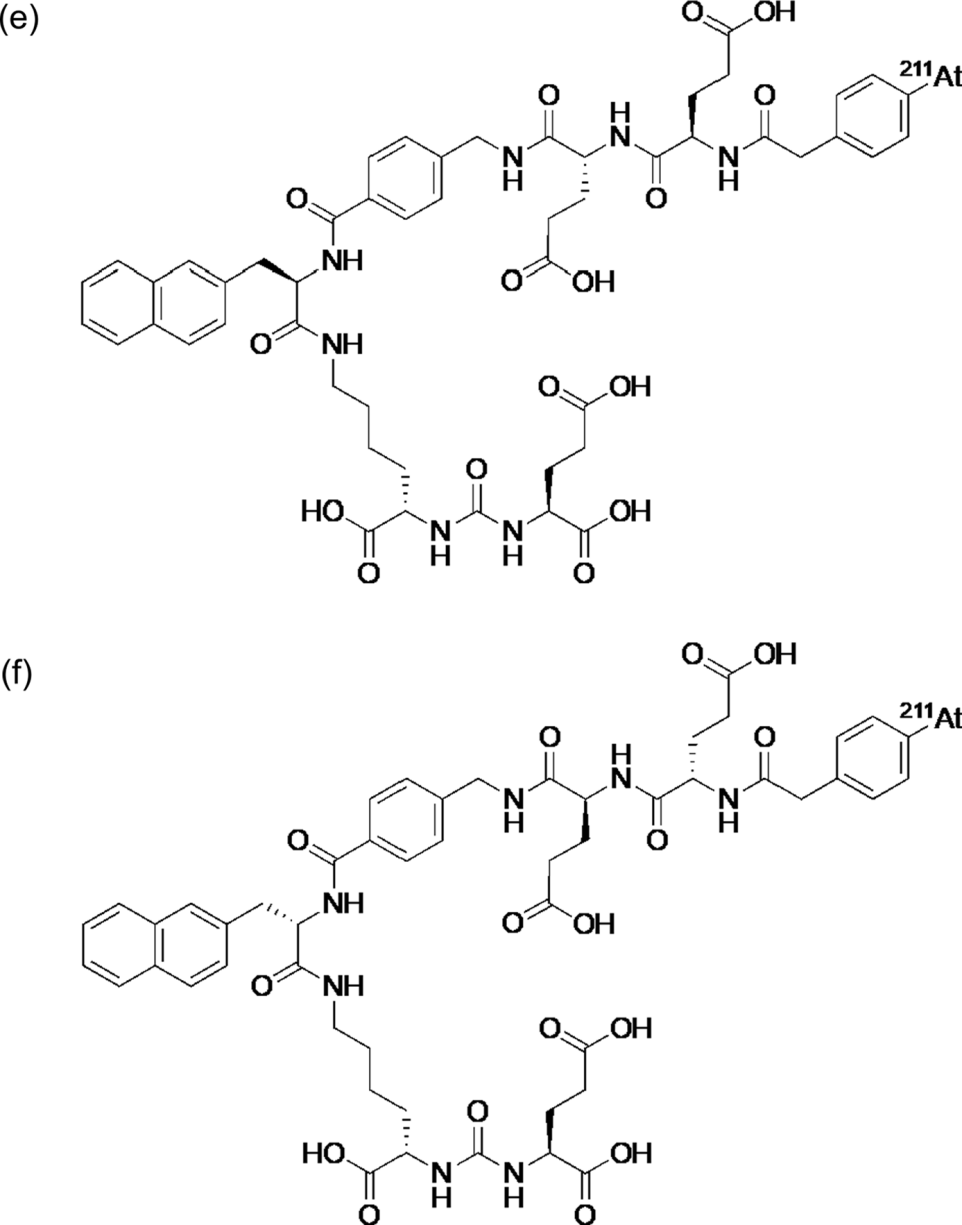
Structures of (2*S*)-2-(3-(1-carboxy-5-(4-^211^At-astatobenzamido)pentyl)ureido)-pentanedioic acid (**a**), ^211^At-**3**-Lu (**b**), [^18^F]F-PSMA-1007 (Pylarify®) (**c**), [^211^At]At-PSMA1 (**d**), [^211^At]At-PSMA5 (**e**), and [^211^At]At-PSMA6 (**f**)

Recently, ^211^At-labeled PSMA ligands were also enthusiastically investigated. First, a simple ^211^At-labeled PSMA ligand, [^211^At]astatobenzoic acid conjugated Glu–urea–Lys, ((2*S*)-2-(3-(1-carboxy-5-(4-^211^At-astatobenzamido)pentyl)ureido)-pentanedioic acid, Fig. 12a), was reported in 2016 [107]: This compound significantly inhibited tumor growth in PSMA + PC3 PIP tumor-bearing mice. The successful results for the above-mentioned ^225^Ac-L1 led to developing an ^211^At-labeled compound (^211^At-**3**-Lu) with a structure similar to that of ^225^Ac-L1 in 2022 (Fig. 12b) [108]. In the ^211^At-labeled compound, nonradioactive Lu coordinated with the DOTA chelator. ^211^At-**3**-Lu showed a pharmacokinetic profile matching the physical half-life of ^211^At and prolonged survival in tumor-bearing animals without off-target toxicity. Meanwhile, other ^211^At-labeled PSMA ligands, [^211^At]At-PSMA1, [^211^At]At-PSMA5, and [^211^At]At-PSMA6 (Fig. 12c–f), as analogs of [^18^F]F-PSMA-1007 (Pylarify^®^), which was approved as second PSMA-targeted PET imaging drug following [^68^Ga]Ga-PSMA-11 by FDA, were also reported in 2023 [109]. Among these ^211^At-labeled PSMA ligands, [^211^At]At-PSMA5 showed the most favorable biodistribution and planar images of [^211^At]At-PSMA5 revealed the tumor tissue at 3 and 24 h postinjection. In therapeutic experiments, [^211^At]At-PSMA5 showed excellent tumor growth suppression in LNCaP tumor-bearing mice without significant body weight loss. These results indicate that ^211^At-labeled PSMA ligands have great potential as agents for TAT to metastatic castration-resistant prostate cancer, and their translational prospective trials are expected shortly.

## Radiolabeled fibroblast-activation protein inhibitors (FAPIs)

The tumor microenvironment is composed of stromal components, with cancer-associated fibroblasts (CAFs) representing the predominant component of the tumor stroma [110]. Cancer cells secrete growth factors that induce the transformation of fibroblasts into CAFs. This activation process leads to high expression of CAF markers such as fibroblast-activating protein (FAP), a type II transmembrane protease known to facilitate tumor growth and metastasis. Moreover, FAP is prominently expressed on the cell surface of activated fibroblasts, as observed in over 90% of epithelial cancers, whereas it is absent in normal adult tissues. Consequently, FAP has been identified as a suitable target for imaging and therapy of various types of tumors [111]. Recently, several clinical trials of ^68^Ga- or ^18^F-labeled FAP inhibitors (FAPIs) PET imaging have been performed [112]. These studies with superior PET images indicate that radiolabeled FAPIs could be an important target for cancer theranostics.

**Fig. 13:**
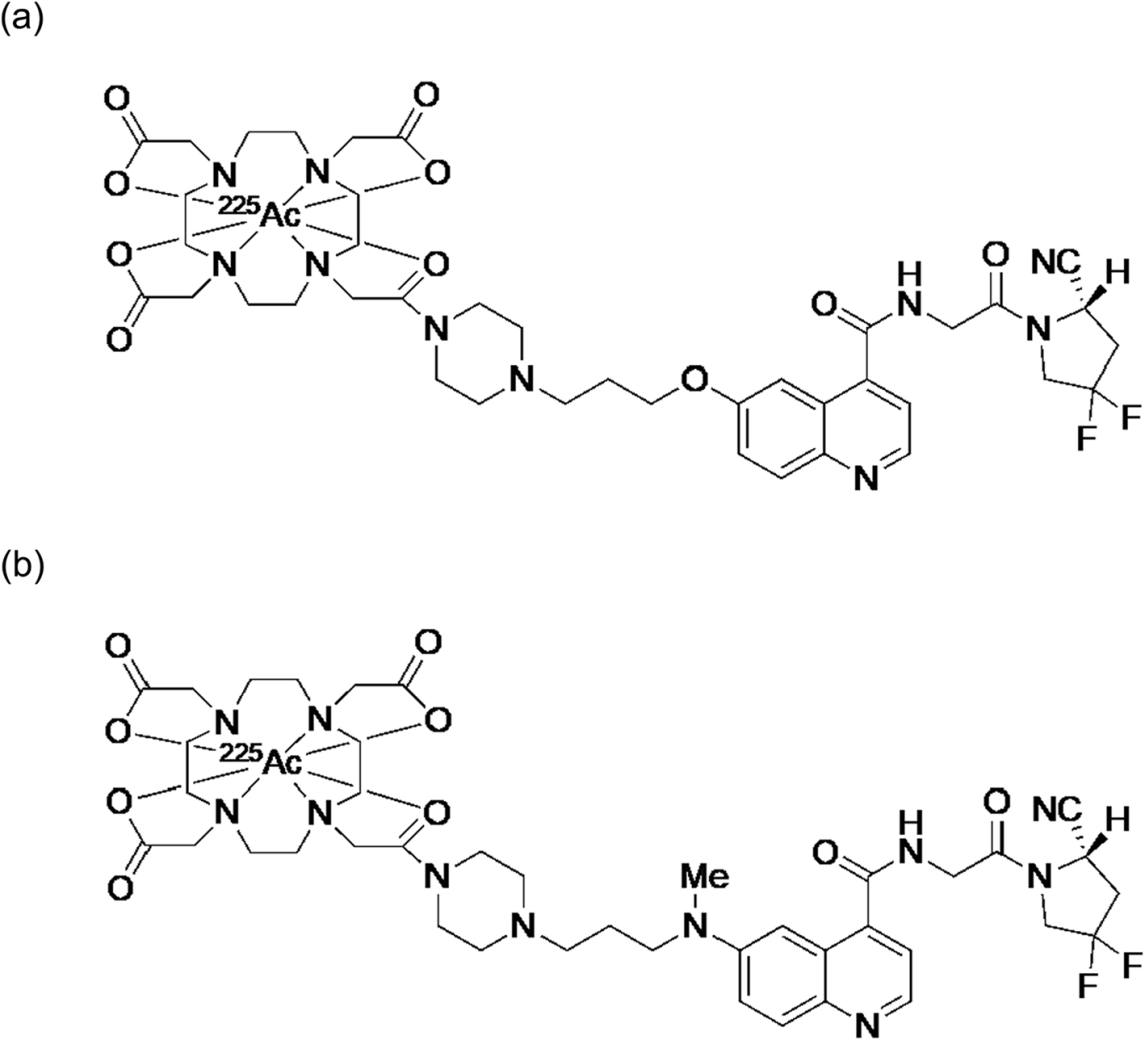
Structures of [^225^Ac]Ac-FAPI-04 (**a**) and [^225^Ac]Ac-FAPI-46 (**b**)

[^225^Ac]Ac-FAPI-04 (Fig. 13a) was first reported in 2020 [113]. [^225^Ac]Ac-FAPI-04 significantly inhibited tumor growth in the PANC-1 tumor-bearing mice compared with that in control mice, without a significant body weight loss. Meanwhile, the clearance of [^225^Ac]Ac-FAPI-04 from the tumor appeared to be too rapid for the physical half-life of ^225^Ac. To improve the tumor retention of the FAPI compounds, FAPI-46 was developed to show a better retention than FAPI-04 [114]. Thus, [^225^Ac]Ac-FAPI-46 was reported in 2022 (Fig. 13b) [115]. However, the therapeutic effects of [^225^Ac]Ac-FAPI-46 were limited. In other words, the tumor-suppressive effects were not significant compared to those in the control group. The improvement in retention was likely insufficient, and the biological half-life of FAPI-46 was too short for the physical half-life of ^225^Ac.

**Fig. 14:**
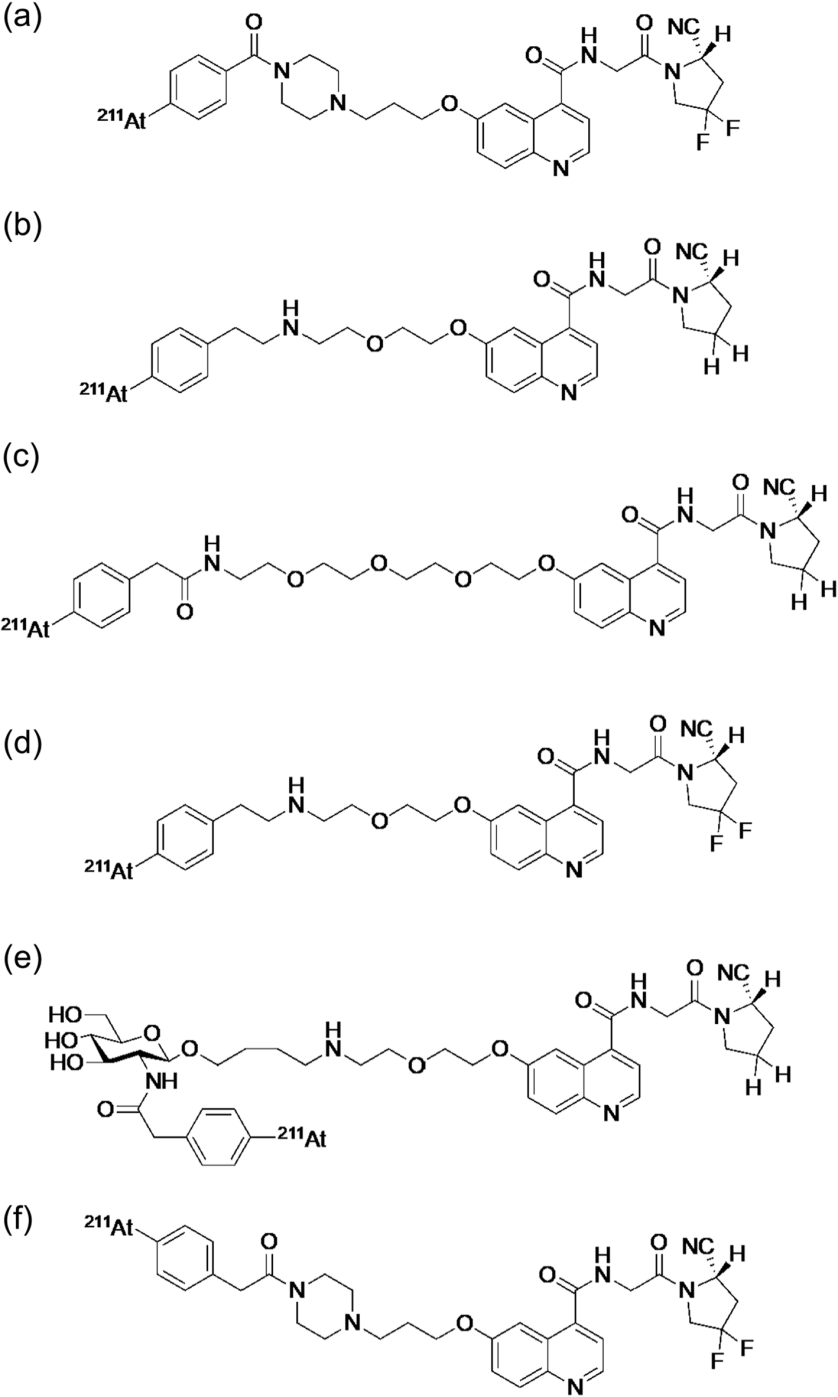
Structures of [^211^At]At-FAPI-04 (**a**), [^211^At]At-FAPI1 (**b**), [^211^At]At-FAPI2 (**c**), [^211^At]At-FAPI3 (**d**), [^211^At]At-FAPI4 (**e**), and [^211^At]At-FAPI5 (**f**)

Compared to ^225^Ac (*t*_1/2_ = 9.9 d), ^211^At (*t*_1/2_ = 7.2 h) could be more favored as an α-emitter radiolabeled with FAPI, which shows fast clearance from the body and tumor. ^211^At-labeled FAPI, [^211^At]At-FAPI-04 (Fig. 14a), was reported in 2022 [116]. [^211^At]At-FAPI-04 showed rapid and specific binding to FAP-positive U87MG cells and dramatically inhibited tumor growth in U87MG tumor-bearing mice in a dose-dependent manner with negligible toxicity. Other ^211^At-labeled FAPIs ([^211^At]At-FAPI1, [^211^At]At-FAPI2, [^211^At]At-FAPI3, [^211^At]At-FAPI4, and [^211^At]At-FAPI5, Fig. 14b–f) with different linkers, polyethylene glycol and piperazine, were reported in 2023 [117]. Among these compounds, [^211^At]At-FAPI1 with a simple PEG linker showed the best properties, and showed higher therapeutic effects than [^211^At]At-FAPI5 with a piperazine linker.

These studies indicate that radiotheranostics containing TAT-targeting FAP in the cancer stroma is effective. Although the detailed therapeutic mechanism is not clear, it could be a new cancer therapeutic strategy in combination with other therapies directly targeting cancer cells.

## Radiolabeled antibodies and their fragments to cancer cell membrane antigens

**Fig. 15:**
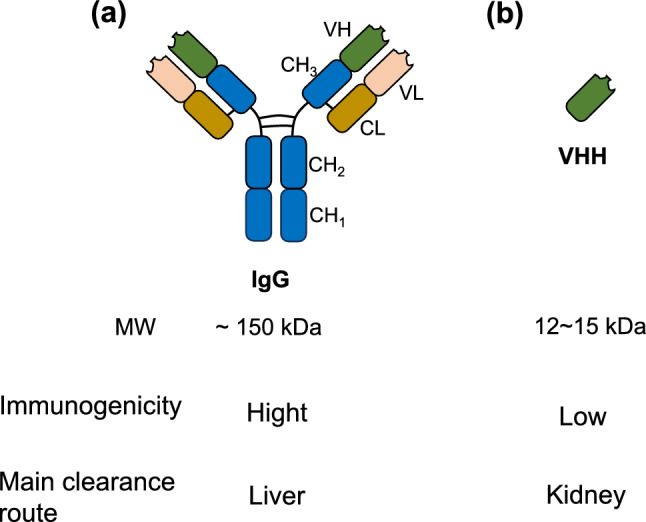
Structures and properties of immunoglobulin G (IgG) antibodies and variable fragments of heavy chain antibodies (VHH). *CH* constant heavy; *VH* variable heavy; *VL* variable light

PET diagnosis using antibodies (also called ImmunoPET) has been developed as a target specific diagnostic tool for various types of cancer [118]. Radiolabeled antibodies could be also applied for radionuclide therapy due to excellent target specificity of antibodies. The anti-CD20 monoclonal antibodies [^131^I]I-tositumomab (Bexxar^®^) and [^90^Y]Y-ibritumomab thiuxetan (Zevalin^®^), labeled with β^−^-emitters, have been used to treat non-Hodgkin’s lymphoma [119]. Immunoglobulin G (IgG) antibodies (*M*_w_ = 150 kDa) have a very high affinity and specificity for their targets (Fig. 15a), making them suitable vectors for TAT [120]. In addition, owing to their long half-life in blood, α-emitters with relatively long half-lives, such as ^225^Ac, are expected to deliver effective antitumor effects. Therefore, IgG-based TAT agents have been primarily developed as ^225^Ac-labeled agents. Fundamental studies were conducted in cells and mice using ^225^Ac-labeled IgG antibodies. Their targets include the human epidermal growth factor receptor 2 (HER2) [121, 122], epidermal growth factor receptor (EGFR) [123], PSMA [124], CD46 [125], CD33 [126, 127], CD20 [128], Carbonic Anhydrase IX [129], Podoplanin [130], and carcinoembryonic antigen [131]. ^211^At-labeled antibodies (and antibody fragments) were also evaluated for CD38 [132], CD123 [133], CD33 [134], CD45 [135], and membrane phosphate transporter protein (NaPi2b). [136] Among these targets, clinical trials have been reported the treatment of acute myeloid leukemia targeting CD33 and ovarian cancer targeting NaPi2b. The main in vivo toxicity observed in both basic and clinical studies is myelotoxicity. Various clinical trials are currently being planned, and although caution must be exercised regarding the side effects, future developments are expected.

Variable fragments of heavy chain antibodies (VHH), smaller in size (12–15 kDa) and less immunogenic than IgG (Fig. 15b), are rapidly cleared from the blood and non-target tissues while maintaining affinity and specificity. Recombinant VHHs can be produced in bulk to reduce costs [137]. This has driven research on VHHs for TAT, including radioligands, not only ^225^Ac but also ^211^At, owing to their shorter blood half-life. Preclinical studies on ^211^At- or ^225^Ac-labeled VHH for HER2 [138, 139], CD20 [140], and 5T2MM idiotypes [141] have also been reported. In biodistribution studies in mice, these VHH-based radioligands reached a plateau in the tumor tissue within about 3–6 h, and showed significantly higher therapeutic efficacy than the non-treated groups. Ertveldt et al. reported that ^225^Ac-anti-CD20 VHH induced systemic antitumor immune responses, suggesting that combination therapy with TAT and tumor immunotherapy may be a promising new cancer treatment tool [140]. However, nephrotoxicity based on the physiological accumulation of VHH has been observed, and caution should be exercised in future clinical applications.

## Radiolabeled nanoparticles for tumor microenvironment

Nanoparticles have gained attention as drug delivery carriers. Nanocarriers are used in nuclear medicine to develop nanoradiopharmaceuticals labeled with γ- or positron-emitter for diagnosis and α- or β^−^-emitter for therapy [142]. ^225^Ac is a promising α-emitter for TAT using nanoparticles since its relatively long half-life (9.9 days) is suitable for the biodistribution of nanoparticles retained in tumors. Liposomes are well-known carriers of active agents, including radiolabeled compounds. Sofou et al. successfully loaded ^225^Ac into liposomes with a high encapsulation efficiency, whereas ^213^Bi, the α-particle-emitting daughter of ^225^Ac, was poorly retained in the liposomes [143]. Maintaining the α-particle-emitting daughters within liposomes during delivery to tumors is important as the cell-killing efficacy of ^225^Ac is partially derived from α-particles emitted from three α-particle-emitting daughters (^221^Fr, ^217^At, and ^213^Bi) generated during ^225^Ac decay (Fig. 1). However, some loss is unavoidable owing to the recoil effect associated with the emission of α-particles from daughters with a recoil distance of 80–90 nm. Increased retention of ^213^Bi has been observed in liposomes with increased particle sizes [143] and in multivesicular liposomes [144]. To enable the therapeutic use of ^225^Ac-containing liposomes, encapsulation efficiency was improved by up to 73% using the active loading method [145]. The ^225^Ac-containing liposomes modified with antibodies or aptamers targeting PSMA show selective accumulation and cytotoxicity in PSMA-expressing cells [146]. In addition, ^225^Ac-labeled liposomes inhibited tumor growth in tumor-bearing mice [147].

Gold nanoparticles [148], LnPO_4_ nanoparticles [149], and calcium core–shell particles [150] have also been reported as ^225^Ac-labeled nanoparticles. Gold nanoparticles were labeled by chelating ^225^Ac via the chelator DOTAGA, which was modified on the surface of the gold nanoparticles. Although daughters were not retained with gold nanoparticles owing to the alpha recoil effect, in vitro and in vivo therapeutic effects were observed. However, LnPO_4_ nanoparticles and calcium core–shell particles doped with ^225^Ac in the core of the nanoparticles were designed to retain ^225^Ac as well as α-particle-emitting daughters. Both nanoparticles exhibited high in vivo stability and biodistribution of ^213^Bi, the last α-particle-emitting daughter, was similar to that of ^225^Ac.

Few studies have used ^211^At-labeled nanoparticles due to the short half-life (7.2 h) of ^211^At. However, ^211^At-labeled gold nanoparticles have been developed as ^211^At can be adsorbed onto gold nanoparticles by simple mixing. The intratumoral injection of ^211^At-labeled gold nanoparticles inhibited tumor growth [151]. The therapeutic effects were dependent on the size of gold nanoparticles; those with a diameter of 5 nm showed the strongest therapeutic effects among those with diameters of 5, 13, 30, and 120 nm. Intravenous injection was also evaluated; ^211^At-labeled gold nanoparticles exhibited potent therapeutic effects in a PANC-1 xenograft model [152].

## Radiolabeled compounds with albumin-binding moiety (ABM) for improved pharmacokinetics and tumor targeting

**Fig. 16:**
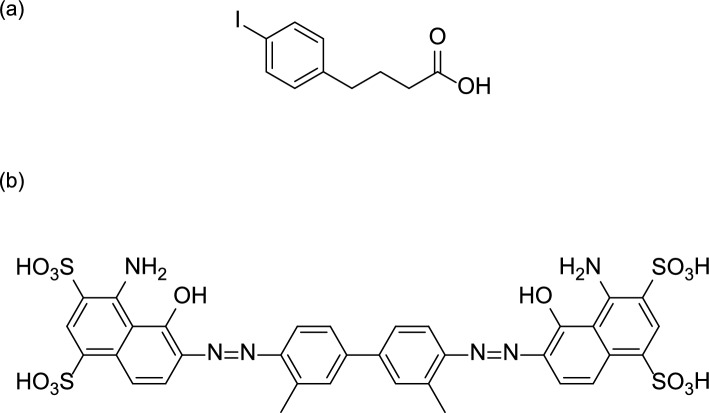
Structures of 4-(4-iodophenyl)butyric acid (**a**), and Evans blue (**b**)

Albumin is the most abundant protein in the body with a biological half-life of 19 days. Albumin contains several distinct binding pockets and is a carrier for endogenous and exogenous compounds such as lipids, hormones, metal ions, and lipophilic drugs. In nuclear imaging, fast clearance of radiolabeled compounds from the blood is generally preferred to achieve a high-tumor-to-blood ratio, which is important for imaging. However, rapid blood clearance can limit tumor uptake, making it difficult to use radiolabeled compounds for therapeutic applications. To overcome these problems, low-molecular-weight albumin-binding molecules such as 4-(4-iodophenyl)butyric acid and Evans blue derivatives have been used for therapeutic applications (Fig. 16) [153, 154]. These albumin binders exhibit non-covalent, reversible interactions with albumin, which extend the in vivo blood circulation time of the radiotracers. Since the dissociation constants of radiotracers against albumin in the low micromolar range are higher than those against targeted receptors in the nanomolar range, increased accumulation in tumors can be achieved by conjugating ABM to conventional radiotracers containing a tumor-targeting moiety.

**Fig. 17:**
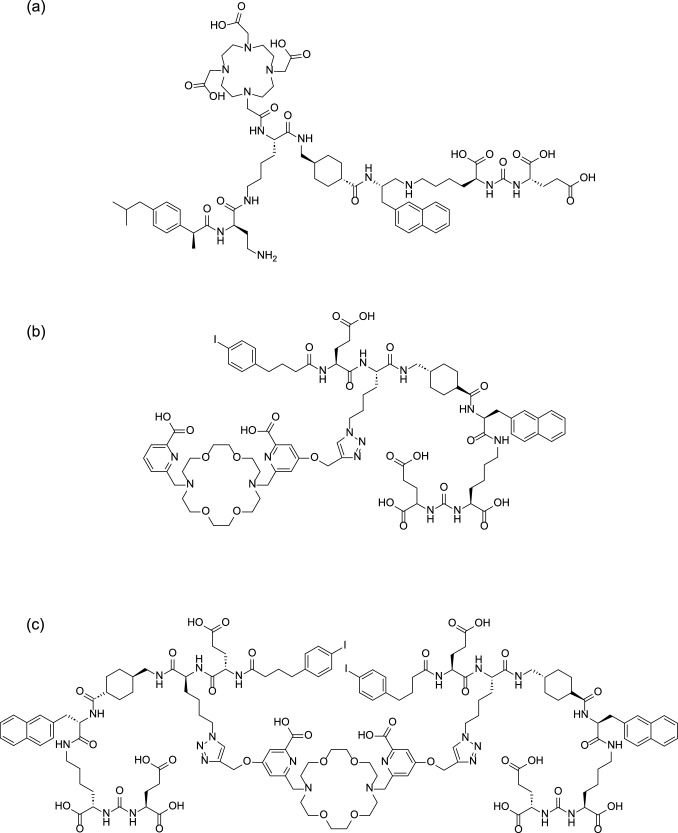
Structures of SibuDAB (**a**), mcp-M-alb-PSMA (**b**), and mcp-d-alb-PSMA (**c**)

Radiotracers containing ABM have been developed as theranostic probes targeting tumor-expressing molecules such as PSMA, SSTR, α_v_β_3_ integrin, folate receptor, glucagon-like peptide-1 receptor and bone [105, 155–157]. There are many reports on the use of DOTA derivatives as chelators for radiometals such as ^67^Ga, ^68^Ga, and ^111^In for diagnostic imaging and ^90^Y and ^177^Lu for therapeutic applications. DOTA derivatives used as chelators of ^225^Ac and ^225^Ac-labeled probes with ABM targeting PSMA (SibuDAB, Fig. 17a) showed increased blood retention, high-tumor accumulation, and potent therapeutic efficacy in PSMA-expressing tumor-bearing mice [158]. The 18-membered macrocycle macropa derivatives have also been described as chelators for ^225^Ac, allowing rapid complexation at room temperature [159]. [^225^Ac]Ac-macropa conjugated compounds with one or two albumin- and PSMA-targeting moieties (mcp-M-alb-PSMA and mcp-d-alb-PSMA, Fig. 17b, c) prolonged the blood circulation time, specifically and highly accumulated in the tumor, and inhibited tumor growth with DNA double-strand break formation [160].

4-(4-Astatophenyl)butyric acid (APBA), in which the iodine in 4-(4-iodophenyl)butyric acid is replaced with astatine, also functions as an ABM, as described in the RGD peptide section [93]. Although only a few reports are present on ^211^At-labeled compounds containing ABM owing to the short half-life of ^211^At, APBA can be applied to other probes with different targeting moieties, which may facilitate the development of ^211^At-labeled compounds containing ABM. The affinity of the probes for albumin was closely related to the kinetic profile of tumor uptake [161]. Lysine-based albumin binders with lower albumin-binding affinities showed higher calculated areas under the curve (AUC) in the tumors among the probes exhibiting albumin-binding affinities from 1.8 to 50 μM. To decrease uptake in normal organs is advantageous for probes containing ABM, such as the kidneys. AUC for the kidney was not affected by the binding affinity of the probes. These results are valuable for designing novel probes containing ABM and will facilitate the development of probes useful for endoradionuclide therapy.

## Conclusion

TAT is a promising treatment in oncology owing to its high cytotoxicity in cancer cells. For TAT, developing probes that deliver α-emitters to the tumor tissues is important. Recently, various probes have been designed targeting molecules specifically expressed in tumors, such as α_v_β_3_ integrin, PSMA, FAP, and SSTR. In preclinical studies using tumor-bearing mice, various probes have exhibited high therapeutic efficacy without serious side effects. Clinical trials are also being conducted, including two in Japan, using [^211^At]NaAt and [^211^At]MABG. The endoradionuclide therapy using α-emitters is expected to be approved and contribute to the treatment of many cancer patients in the near future.

## Data Availability

There are no need to include a data availability statement in this article.

## Abbreviations

TAT: Targeted alpha therapy
LET: Linear energy transfer
DOTA: 1,4,7,10-Tetraazacyclododecane-1,4,7,10-tetra-acetic acid
EC: Electron capture
SPECT: Single photon emission computed tomography
NIS: Sodium/iodide symporter
NE: Norepinephrine
MIBG: *m*-Iodobenzylguanidine
MABG: *m*-Astatobenzylguanidine
NET: Neuroendocrine tumor
SSTR: Somatostatin receptor
FDA: Food and Drug Administration
PET: Positron emission tomography
ABM: Albumin-binding moiety
CAF: Cancer-associated fibroblast
FAP: Fibroblast-activating protein
FAPI: Fibroblast-activating protein inhibitor
IgG: Immunoglobulin G
APBA: 4-(4-Astatophenyl)-butyric acid
AUC: Areas under the curves
