# Correction: Recent advances in the development of ^225^Ac- and ^211^At-labeled radioligands for radiotheranostics

**DOI:** 10.1007/s44211-025-00763-3

**Published:** 2025-04-09

**Authors:** Masayuki Munekane, Takeshi Fuchigami, Kazuma Ogawa

**Affiliations:** 1 Graduate School of Medical Sciences, Kanazawa University, Kakuma-Machi, Kanazawa, Ishikawa 920-1192 Japan; 2 Institute for Frontier Science Initiative, Kanazawa University, Kakuma-Machi, Kanazawa, Ishikawa 920-1192 Japan

**Correction: Analytical Sciences (2024) 40:803–826** 10.1007/s44211-024-00514-w

In Fig. 2 of this article the half-life of 211Po should have been 0.516 s, not 7.214 h. The figure should have appeared as shown below.
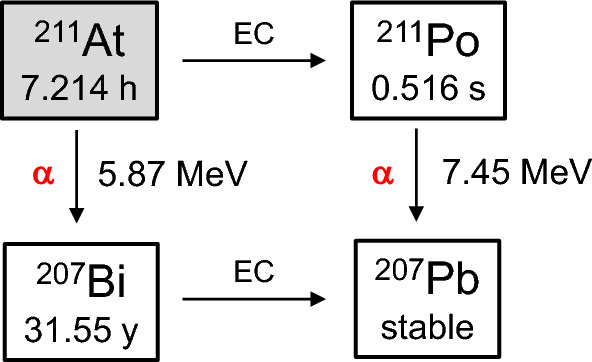


The original article has been corrected.

